# The Remove-the-Mask Open-Source head and neck Surface-Guided radiation therapy system

**DOI:** 10.1016/j.phro.2024.100541

**Published:** 2024-01-29

**Authors:** Youssef Ben Bouchta, Mark Gardner, Chandrima Sengupta, Julia Johnson, Paul Keall

**Affiliations:** The University of Sydney, Camperdown, NSW 2050, Australia

**Keywords:** Surface-Guided Radiotherapy, Intra-Fraction Motion Monitoring, Open-Source, Head And Neck Radiotherapy, Image-Guided Radiotherapy

## Abstract

**Background and Purpose:**

Surface Guided Radiotherapy (SGRT) for head and neck radiotherapy is challenging as obstructions are common and non-rigid facial motion can compromise surface accuracy. The purpose of this work was to develop and benchmark the Remove the Mask (RtM) SGRT system, an open-source system especially designed to address the challenges faced in radiotherapy of head and neck cancer.

**Materials and Methods:**

The accuracy of the RtM SGRT system was benchmarked using a head phantom positioned on a robotic motion platform capable of sub-millimetre accuracy which was used to induce unidirectional shifts and to reproduce three real head motion traces. We also assessed the accuracy of the system in ten humans volunteers. The ground truth motion of the volunteers was obtained using a commercial motion capture system with an accuracy < 0.3 mm.

**Results:**

The mean tracking error of the RtM SGRT system for the ten volunteers was of −0.1 ± 0.4 mm −0.6 ± 0.6 mm and 0.3 ± 0.2 mm, and 0.0 ± 0.2^°^ 0.0 ± 0.1^°^ and 0.0 ± 0.2^°^ for translations and rotations along the left–right, superior-inferior and anterior-posterior axes respectively and we also found similar results in measurements with the head phantom. Forced facial motion was associated with lower tracking accuracy. The RtM SGRT system achieved submillimetre accuracy.

**Conclusion:**

The RtM SGRT system is a low-cost, easy to build and open-source SGRT system that can achieve an accuracy that meets international commissioning guidelines. Its open-source and modular design allows for the development and easy translation of novel surface tracking techniques.

## Highlights

• Open-source head and neck SGRT system developed to enable novel SGRT algorithm development.
• Achieves a sub-degree and submillimetre surface tracking accuracy.
• Allow easy access to raw surface images with high temporal resolution.
• Easy to replicate and use in both laboratory and a clinical settings.

## 1 Introduction

Treatment with radiotherapy (RT) is indicated for the majority of head and neck (H&N) cancer patients [1], [2]. Radiotherapy drastically improves overall survival in H&N cancer patients; however, safe and effective H&N RT requires a higher accuracy than for most other treatment sites [1], [2], [3], [4], [5], [6], [7], [8]. This need for highly accurate radiation delivery stems from the necessity of generating steep dose gradients, and the radiation sensitivity of organs-at-risk (OARs) such as the brainstem, spinal cord, optical nerve, etc. and their proximity to the treated area [2], [9].

To achieve the high level of precision needed, H&N RT treatments combine pre-treatment imaging to correct for day-to-day changes in internal anatomy and strict patient immobilisation with a thermoplastic mask [9], [10], [11]. The use of such strict immobilisation causes up to 50 % of patients to experience claustrophobia and mask-related anxiety [12], [13], [14], [15], which can be severe enough to reduce treatment adherence or trigger treatment refusal [16]. Mask related anxiety can also lead to long-term mental health impacts such as symptoms of post-traumatic stress and recurrent nightmares [17], [18]. Immobilisation masks also partially negate the skin-sparing effect of megavoltage RT and increase skin dose by up to 18 % [19], [20] on average for coplanar RT and up to 58 % for non-coplanar, non-isocentric beam delivery [21]. Furthermore, the level of immobilisation that thermoplastic masks can achieve degrades with weight-loss induced changes in head anatomy [22], [23], [24]. These changes can only be corrected through remaking the mask, a process that requires rescanning the patient and replanning, which negatively impact clinical workflow and can delay treatment. Ahn *et al*. found that 65 % of H&N patients in their study required a replanning at some point during their treatment, with anatomical changes leading to clinically significant tumour underdosing in 61 % of patients and to the spinal cord dose exceeding the safe threshold in 30 % of patients [25].

Open-face masks provide an alternative to thermoplastic masks that may reduce the incidence of mask-related anxiety and claustrophobia and provides similar level of immobilisation to closed masks [26], although patient motion exceeding clinical thresholds has been associated with their use [27]. As a result, open-face masks are often combined with surface guided radiotherapy (SGRT) to accelerate patient positioning and provide motion monitoring in real-time without exposing the patient to harmful radiation [28].

While SGRT’s usefulness in ensuring accurate treatment delivery for patients treated with open-face masks is undisputed, current SGRT systems struggle to accurately track non-rigid motion. Facial motion has been shown to reduce surface tracking accuracy [29], [30] and changes in facial expression can even generate erroneous positioning correction for patients treated with an open-face mask [31]. Additionally, ceiling-mounted SGRT systems are subject to field-of-view obstructions by C-arm linacs gantry and kV imagers, something that occurs most commonly in H&N treatment due to the treatment geometry [28]. Furthermore, the shape of the human face lends itself to self-occlusion, a type of obstruction that occurs when a section of the imaged surface obstructs another section (e.g., the nose can hide the forehead or cheeks from view). Current SGRT systems solve this issue by using multiple surface imaging sensors located around the treatment room with overlapping field of view however, single sensor obstructions have been shown to reduce surface tracking accuracy [32] and the combination of self-obstruction and gantry obstruction can result in SGRT systems losing sight of the H&N during treatment delivery. The deployment of the kV imager, such as when acquiring a CBCT or for real-time Image-Guided Radiotherapy (IGRT) and large couch tilts increase the probability that a complete obstruction event will occur.

Solving those issues and creating a system that can accurately track H&N motion when faced with changes in facial expression during a RT treatment requires the development of novel surface tracking algorithms to handle surface deformation. In this study, we present the Remove the Mask (RtM) open-source H&N SGRT system. We aim to benchmark the accuracy of this system and to assess its accuracy and the factors impacting its accuracy for immobilisation-free H&N surface imaging in a radiotherapy setting.

## 2 Methods and materials

**Fig. 1:**
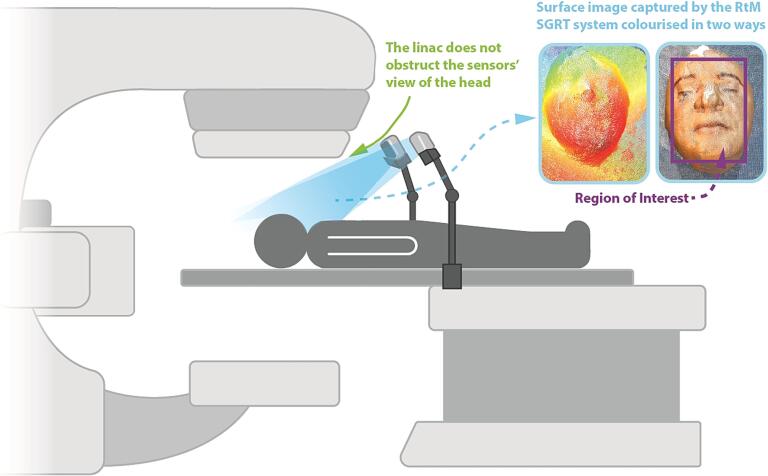
Rendition of how the RtM SGRT system would be used in the clinic showing the depth sensors position, the lack of obstruction from a C-arm linac and a surface image of a phantom captured by the RtM SGRT system colourised by distance to the camera (left) and with real-world colour (right). A typical ROI is shown superimposed on the real-world coloured image.

The RtM SGRT system’s hardware consisted of two depth sensor cameras (Intel RealSense D415) used to image the surface of the patient. The sensors were positioned on each side of the patient, about 30 cm above the abdomen as shown in Fig. 1. This setup was chosen as it allowed the sensors to have an unimpeded view of the head, neck, and shoulders of the patient regardless of gantry angle and of whether the kV imager had been deployed on a C-arm linac. The sensors each contained 2 infrared cameras that were factory calibrated as a stereo depth camera, and a RGB camera that is factory calibrated to allow for colourisation of the surface data. Carfagni *et al.* have published a detailed description and characterisation of the image acquisition process of the Intel RealSense D415 sensor [33].

To control the surface imaging sensors and accomplish real-time surface tracking for the RtM system, we developed the Surface Guided Radiotherapy Toolkit in Python (SpyGRT) which we have made publicly available on GitHub under an MIT license [34]. SpyGRT is a modular Python package allowed real-time surface data acquisition, processing and recording as well as in-depth data analysis of recorded data. Additionally, it also featured a calibration algorithm that allowed for verification that the frame of reference of the surface imaging system matched that of the treatment room.

The RtM SGRT system was used to capture, record and analyse the data in all experiment in this study. The data was captured at a frame rate of 30 fps however, due speed limits on data transfer and on writing to memory, the actual average frame rate of the recorded data was 15 fps. The average speed at which the RtM SGRT system was able to perform surface tracking was 4.5 fps. The quoted computational speed values were obtained when data capture and surface tracking were performed on a computer equipped with 32 GB DDR4 RAM and an NVIDIA RTX A5000 GPU. The surface motion data presented in this study was obtained during post-capture analysis to maximize the sampling rate of the measured motion.

### 2.1 Surface tracking algorithm

Like most SGRT systems, the RtM system tracks surfaces by performing a registration between a reference surface (which can either be acquired live, or imported from a prior session or the planning CT) and the most recently acquired surface image (the target surface). To increase the speed of the registration and remove non-patient points (such as the headrest) from the surface, all points outside of a pre-defined region-of-interest (ROI) are cropped from the target surface prior to the registration.

A feature of the RtM system is that the user-defined ROI is only used for the first frame in the tracking sequence. For each subsequent frame, the motion estimation for the previous frame is used to shift the ROI so that its location with respect to the patient is unchanged and therefore ensures that the target surface contains the same section of the patient’s head in each frame. The motion estimation from the previous frame is used as the starting point for the surface registration of the current frame.

The registration algorithm used by the RtM system is the Open3D [35] implementation of the point-to-plane Iterative Closest Point (ICP) algorithm [36]. This algorithm minimises the cost function shown in Eq. (1) where p and q are corresponding points on the target and reference surface respectively, T is the 6 degrees-of-freedom (DoF) rigid transformation between the target and reference surface, $n_{p}$ is the normal of the target surface at point p, and $w_{\mathrm{pq}}$ is a weighting factor for the p*,* q corresponding pair and is calculated using the generalised adaptive loss function described by Barron [37].

(1) $$Cost\left(T\right)=\sum_{\left(p,q\right)\in Corr}{w_{\mathrm{pq}}\left[\left(p-Tq\right)∙n_{p}\right]}^{2}$$

The results of the registration are converted into a translation vector and a set of Euler angles which can be used for couch corrections. To improve tracking accuracy and preserve a high temporal resolution, the optimisation is performed in a 5-step rough-to-fine process. The first iteration of the registration is performed with the surfaces downsampled to 0.2 points per square centimeter, and the last iteration with 100 points per square centimeter.

### 2.2 Benchmarking the accuracy through phantom experiments

**Fig. 2:**
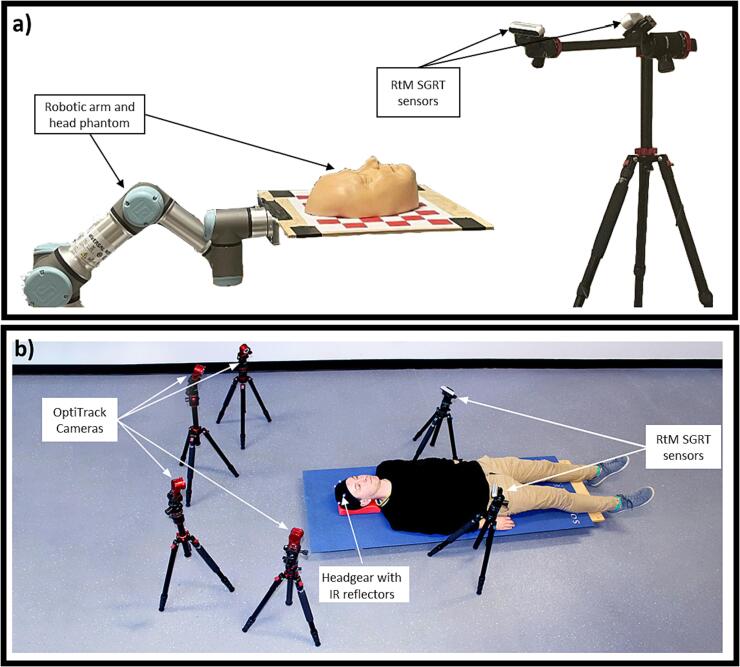
Depiction of the experimental setup used for the phantom measurements (a) and of the VISION study set-up (b).

A phantom experiment was designed to test the accuracy of the RtM SGRT system using a UR16, a robotic arm capable of 6 DoF motion with < 0.2 mm and < 0.6° accuracy and which has previously been validated for use in real-time tumour tracking QA [38], [39]. The phantom experiment aimed at quantifying the accuracy of the RtM SGRT system in a more controlled setting where all motion was rigid. A head phantom was secured to the robot and moved at fixed intervals of 1 mm from −5 to 5 mm and 1 deg from −5 deg to 5 deg one axis at a time for each translation and rotation axis. We also reproduced three real head and neck motions traces to evaluate the impact of surface deformation on the accuracy of our system. Fig. 2a shows the experimental setup.The tracking error for phantom experiments was defined as the difference between the motion measured by the RtM SGRT system and the motion trace given as an input to the UR16.

### 2.3 Evaluating accuracy for human participants: VISION study

The Visual Investigation of Surface Imaging for Oncological Needs (VISION) study is a human volunteer study approved by our institutional research ethics committee with the aim of acquiring surface images under similar conditions as that encountered in the clinic and to test the accuracy of the RtM SGRT system on human subjects. All participants in the VISION study provided informed consent prior to their participation.

During the study, participants were asked to lay down on a couch with their head resting on a neck support similar to those used in the clinic. Participants were asked to lay as still as possible for 10 min, and then asked to perform a series of head and facial motion: yawn, smile, slowly shake their head, frown, and swallow 5 times. Surface images were recorded throughout the entire study session with the RtM system, and the initial depth sensor temperature was measured using a temperature sensor embedded on the depth sensor chip. Initial sensor temperature was chosen for analysis as it indicates the extent to which the depth sensors were warmed up prior to the imaging session. The ground truth motion was the motion of 4 infrared reflectors located on a headgear worn by the participants and was captured with the OptiTrack (NaturalPoint, Corvallis, Or USA) commercial motion capture system which has a quoted accuracy of < 0.3 mm and < 0.1 deg. OptiTrack was independently validated using the UR16 (Universal Robots, Novi, MI, USA) robotic arm described in Section 2.3. The VISION study setup is shown in Fig. 2b. The tracking error when tracking human volunteer was defined as the difference between the motion measured by the RtM SGRT system and that measured by the OptiTrack system.

For each participant, the initial ROI was defined as the region from the boundary of the forehead and hairline to just below the chin. For subsequent frames, the ROI was adapted as described in Section 2.1. In this work, we present data from 10 VISION study participants, 5 male and 5 female, and compare the motion recorded by the RtM SGRT system with that recording by OptiTrack.

A multiple linear regression was performed to determine the impact of gender and initial sensor temperature on the error in the measured total displacement vector. A *t*-test was used to determine whether the coefficients of the regression were different from 0 and the statistical significance of those results.

## 3 Results

### 3.1 Benchmarking of the accuracy of the RtM SGRT system

**Fig. 3:**
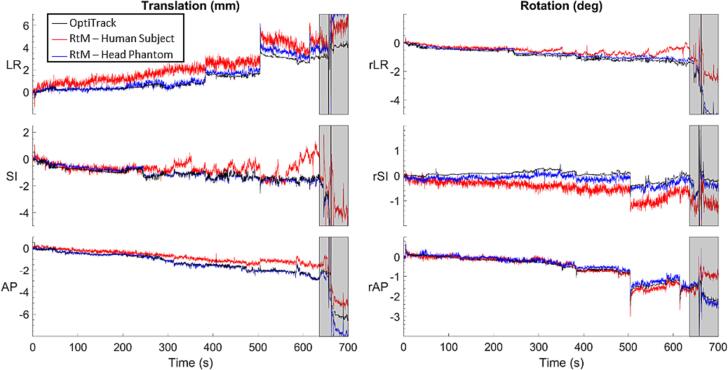
Sample translational (left) and rotational (right) motion trace of a VISION participant. The ground truth motion measured by OptiTrack is shown in black, the RtM measured motion is shown in red and the RtM measured motion when the motion trace was replicated with the head phantom on the UR16 is shown in blue. The grey box at the end of each plot shows the section of the recording when the volunteer was asked to perform different facial motion. (For interpretation of the references to colour in this figure legend, the reader is referred to the web version of this article.)

**Fig. 4:**
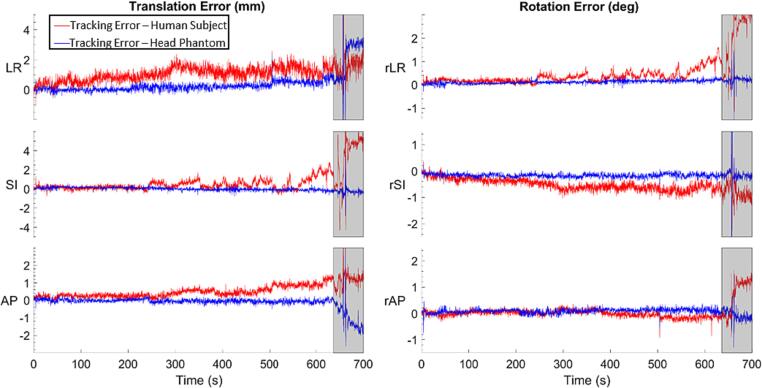
Sample Translational (left) and rotational (right) tracking error of the RtM system when tracking a VISION participant (red) when tracking the head phantom while the motion trace was replicated on the UR16 (blue). The grey box at the end of each plot shows the section of the recording when the volunteer was asked to perform different facial motion. The data in this figure is from the same participant as for the data in Fig. 3. (For interpretation of the references to colour in this figure legend, the reader is referred to the web version of this article.)

**Fig. 5:**
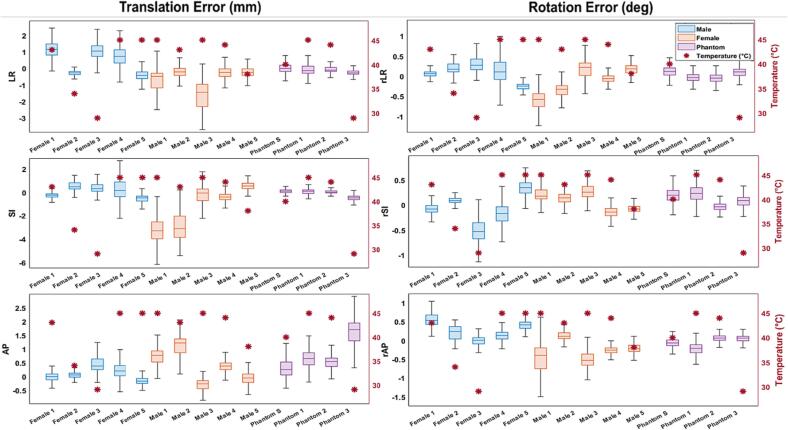
Boxplot of the tracking error of the RtM SGRT system for each rotational and translational axes for VISION partcipants and phantom traces with initial sensor temperature overlayed. The lower, middle and upper bound of each box represents the 25th, 50th and 75th percentile of the tracking error, respectively and the whiskers length is 1.5 times the interquartile range. The Phantom S box represents the tracking error when tracking single axis motion, whereas the Phantom 1–3 boxes represents the tracking error for the head phantom on the UR16 replicating a human subject motion trace.

The tracking error (mean ± standard deviation) of the RtM system in tracking single axis translations and rotations was 0.0 ± 0.4 mm, 0.1 ± 0.3 mm and 0.3 ± 0.4 mm, and 0.1 ± 0.2 deg, 0.2 ± 0.2 deg and 0.0 ± 0.2 deg along the left–right (LR), superior-inferior (SI) and anterior-posterior (AP) axes respectively. For H&N motion traces reproduced on the UR16, the mean tracking error was −0.1 ± 0.3 mm, −0.2 ± 0.2 mm and 0.9 ± 0.4 mm, and 0.0 ± 0.1 deg, 0.1 ± 0.1 deg and 0.0 ± 0.1 deg for translations and rotations along the LR, SI and AP axes respectively. Fig. 3 shows one of the H&N motion traces reproduced on the UR16 and the motion measured by the RtM system. Fig. 4 shows the tracking error for that same motion trace. Error metrics and initial sensor temperature for all motion traces are shown in Fig. 5.

### 3.2 Accuracy of the RtM SGRT system in human subjects

The tracking error when tracking human volunteer was defined as the difference between the motion measured by the RtM SGRT system and that measured by the OptiTrack system. The surface tracking error of the RtM system on human subject was of −0.1 ± 0.4 mm −0.6 ± 0.6 mm and 0.3 ± 0.2 mm, and 0.0 ± 0.2 deg, 0.0 ± 0.1 deg and 0.0 ± 0.2 deg for translations and rotations along the LR, SI and AP axes respectively. Fig. 3 shows the recorded motion data for one of the participants. Fig. 4 shows the tracking error for that same motion trace.

The linear multiple regression showed no statistically significant correlation between gender and tracking accuracy (p = 0.37) but found that a low initial sensor temperature was correlated with worse tracking accuracy (p = 0.02).

**Fig. 6:**
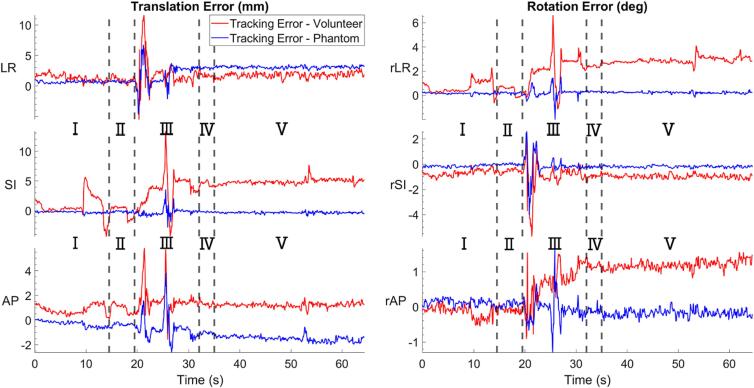
Close up of the grey shaded portion of each plot in Fig. 4 showing the translational (left) and rotational (right) tracking error of the RtM system when tracking a VISION participant (red) and when tracking the head phantom while the motion trace was replicated on the UR16 (blue). The vertical line indicates where one facial motion ends and the next begin. The different motions where a yawn(Ⅰ), a smile(Ⅱ), a head shake (Ⅲ), a frown (Ⅳ) and 5 successive swallows(Ⅴ). (For interpretation of the references to colour in this figure legend, the reader is referred to the web version of this article.)

We found that surface deformation from changes due to changing facial expression reduce the surface tracking accuracy. Fig. 6 compares the tracking error when a participant was instructed to perform forced facial motion and when the same head motion was reproduced on a rigid phantom.

## 4 Discussion

The RtM SGRT system is a novel H&N surface-guidance system that can be built with readily available consumer electronics and based on an open-source surface guidance code. In this study, the tracking accuracy of this system for both phantom and human subject was < 1 mm for translations and < 1 deg for rotations in both phantom measurements and in human subjects. This is in line with the published ESTRO guidelines on SGRT [32] and is comparable to what other studies have found for clinical SGRT systems [40], [41]. An important factor when evaluating the true accuracy of the RtM SGRT system is that the accuracy of the OptiTrack system which was used as ground truth is < 0.3 mm, which is similar in magnitude to the accuracy measured for the RtM SGRT system. As such, the true accuracy of the RtM SGRT system might differ slightly from the reported tracking error in this study.

The RtM SGRT system is a research H&N SGRT system that can be used to capture large datasets of surface images with high temporal resolution, and to facilitate the development, implementation, and testing of novel H&N SGRT techniques and algorithms. The couch mounted design of the RtM SGRT system bypasses the issue of occlusions from the gantry and kV imagers and allows easy installation and does not necessitate permanent modifications to the treatment vault which is ideal for a research system as it minimizes the impact on clinical practice when not in use. Furthermore, it also ensures that the tracking accuracy is not impacted by couch rotations, something which has been shown to reduce the accuracy of ceiling mounted SGRT systems [42]. However, this design comes with the disadvantage of being unable to detect or verify couch motion since the SGRT sensors move with the couch. Additionally, the SGRT system would be dependant on the couch’s own internal position monitoring to maintain isocenter alignment after couch corrections.

A major source of uncertainty in SGRT motion monitoring for H&N RT treatments with open-face masks is the reduced accuracy of surface tracking in the presence of non-rigid surface deformations induced by facial movements [30], [31]. A solution to this issue will require the development of SGRT algorithms which are more robust to non-rigid motion. C-RAD's Catalyst HD has implemented a non-rigid motion tracking [43], but previous studies have shown that surface deformations still had significant impacts on its tracking accuracy [31].

Another limitation of this study is that we did not assess the motion of the internal anatomy given the observed surface motion. Given the presence of deformation in the volunteer data, it is possible that the measured surface motion was not representative of the internal motion. A similar effect As a result, the tracking error in this study represents the difference between the ground truth rigid motion and the observed motion rather than an error in the target position estimation, the more relevant quantity in a clinical setting. This effect would be particularly relevant to deeper target which are located further away from the imaged surface.

Similarly, since the point-to-plane registration outputs a 3D transformation rather than an independent translation and rotation, it is not fully feasible to differentiate between translational and rotational accuracy. Since the centre of origin of the SGRT system does not coincide with the centre of rotation of a person’s head, a small error in rotation could cause a corresponding error in translation. The magnitude of this effect would increase with distance between the origin of the SGRT system and the centre of rotation.

In its current phase of development, ease of adoption of the RtM SGRT system in a clinical setting is limited by the lack of a simple user interface and the lack of an easy to build couch mount for the surface imaging sensors. Future work will focus on improving ease of adoption and on using the RtM system to develop novel surface tracking algorithm that improve the robustness and accuracy of SGRT for H&N. We also plan to add functionalities that will allow the RtM SGRT system to be combined with fluoroscopic x-ray motion tracking modalities as recent studies have shown that using surface data in combination with x-ray imaging improves real-time tumour localisation accuracy compared to either modality alone [44].

The RtM system, a low-cost, adaptable and open-source SGRT system, lowers the barrier to entry to SGRT research and advances SGRT technology. To our knowledge, it is the only publicly available SGRT system that can capture surface images at high frequency in a clinical radiotherapy setting and has the flexibility to test new SGRT algorithms. In addition, while the focus of this work was on H&N RT applications, SpyGRT, the open-source software that was developed for the RtM SGRT system, does not depend on any H&N specific parameter, and can therefore be used to develop novel SGRT solutions and techniques for any RT treatment site. We believe that the RtM SGRT system will allow the radiation oncology community to develop SGRT applications to harness the full potential of surface imaging in improving RT treatments. While it is currently limited to rigid-motion tracking, we hope to develop the RtM system into a SGRT system capable of maintaining its accuracy in the presence of non-rigid facial deformation and that can enable safe and effective mask-free H&N RT when combined with real-time IGRT.

In conclusion, we have shown that the accuracy of the RtM SGRT meets both the AAPM and the ESTRO-ACROP international commissioning guidelines for SGRT systems. Furthermore, the RtM system is the only publicly available SGRT system that can capture the high temporal resolution surface data needed for the development of new SGRT techniques and algorithms while also allowing easy implementation and testing of novel surface tracking algorithms.

## CRediT authorship contribution statement

**Youssef Ben Bouchta:** Conceptualization, Methodology, Software, Investigation, Formal analysis, Writing – original draft, Project administration. **Mark Gardner:** Conceptualization, Writing – review & editing. **Chandrima Sengupta:** Investigation, Resources, Writing – review & editing. **Julia Johnson:** Visualization. **Paul Keall:** Supervision, Conceptualization, Funding acquisition, Writing – review & editing.

## Declaration of competing interest

The authors declare that they have no known competing financial interests or personal relationships that could have appeared to influence the work reported in this paper.

## References

[1] Mendenhall W.M., Hinerman R.W., Amdur R.J., Malyapa R.S., Lansford C.D., Werning J.W. (2006). Postoperative radiotherapy for squamous cell carcinoma of the head and neck. Clin Med Res.

[2] Anderson G, Ebadi M, Vo K, Novak J, Govindarajan A, Amini A. An updated review on head and neck cancer treatment with radiation therapy. Cancers (Basel) 2021;13. 10.3390/cancers13194912. 10.3390/cancers13194912 PMC8508236 34638398

[3] Navran A., Heemsbergen W., Janssen T., Hamming-Vrieze O., Jonker M., Zuur C. (2019). The impact of margin reduction on outcome and toxicity in head and neck cancer patients treated with image-guided volumetric modulated arc therapy (VMAT). Radiother Oncol.

[4] Rosenthal D.I., Lewin J.S., Eisbruch A. (2006). Prevention and treatment of dysphagia and aspiration after chemoradiation for head and neck cancer. J Clin Oncol.

[5] Goel A.N., Lee J.T., Wang M.B., Suh J.D. (2020). Treatment delays in surgically managed sinonasal cancer and association with survival. Laryngoscope.

[6] Nishimura H., Sasaki R., Yoshida K., Miyawaki D., Okamoto Y., Kiyota N. (2012). Radiotherapy for Stage I or II hypopharyngeal carcinoma. J Radiat Res.

[7] Gamez M.E., Blakaj A., Zoller W., Bonomi M., Blakaj D.M. (2020). Emerging concepts and novel strategies in radiation therapy for laryngeal cancer management. Cancers (Basel).

[8] Lee A.W.M., Sze W.M., Au J.S.K., Leung S.F., Leung T.W., Chua D.T.T. (2005). Treatment results for nasopharyngeal carcinoma in the modern era: the Hong Kong experience. Int J Radiat Oncol Biol Phys.

[9] Kearney M., Coffey M., Leong A. (2020). A review of Image Guided Radiation Therapy in head and neck cancer from 2009–201 – Best Practice Recommendations for RTTs in the Clinic. Tech Innov Patient Support Radiat Oncol.

[10] Schwarz M., Giske K., Stoll A., Nill S., Huber P.E., Debus J. (2012). IGRT versus non-IGRT for postoperative head-and-neck IMRT patients: dosimetric consequences arising from a PTV margin reduction. Radiat Oncol.

[11] Yu Y., Michaud A.L., Sreeraman R., Liu T., Purdy J.A., Chen A.M. (2014). Comparison of daily versus nondaily image-guided radiotherapy protocols for patients treated with intensity-modulated radiotherapy for head and neck cancer. Head Neck.

[12] Nixon J.L., Cartmill B., Turner J., Pigott A.E., Brown E., Wall L.R. (2018). Exploring the prevalence and experience of mask anxiety for the person with head and neck cancer undergoing radiotherapy. J Med Radiat Sci.

[13] Nixon J.L., Brown B., Pigott A.E., Turner J., Brown E., Bernard A. (2019). A prospective examination of mask anxiety during radiotherapy for head and neck cancer and patient perceptions of management strategies. J Med Radiat Sci.

[14] Oultram S., Findlay N., Clover K., Cross L., Ponman L., Adams C. (2012). A comparison between patient self-report and radiation therapists’ ability to identify anxiety and distress in head and neck cancer patients requiring immobilization for radiation therapy. J Radiother Pract.

[15] Sharp L., Lewin F., Johansson H., Payne D., Gerhardsson A., Rutqvist L.E. (2005). Randomized trial on two types of thermoplastic masks for patient immobilization during radiation therapy for head-and-neck cancer. Int J Radiat Oncol Biol Phys.

[16] Clover K., Oultram S., Adams C., Cross L., Findlay N., Ponman L. (2011). Disruption to radiation therapy sessions due to anxiety among patients receiving radiation therapy to the head and neck area can be predicted using patient self-report measures. Psychooncology.

[17] Molassiotis A., Rogers M. (2012). Symptom experience and regaining normality in the first year following a diagnosis of head and neck cancer: A qualitative longitudinal study. Palliat Support Care.

[18] Keast R., Sundaresan P., Burns M., Butow P.N., Dhillon H.M. (2020). Exploring head and neck cancer patients’ experiences with radiation therapy immobilisation masks: a qualitative study. Eur J Cancer Care (Engl).

[19] Lee N., Chuang C., Quivey J.M., Phillips T.L., Akazawa P., Verhey L.J. (2002). Skin toxicity due to intensity-modulated radiotherapy for head-and-neck carcinoma. Int J Radiat Oncol Biol Phys.

[20] Nagpal P., Pruthi S., Shanmugan P., Chinnakari P., Pandey M., Singh H. (2021). Cancer Ther Oncol Int J Does thermoplastic mask alleivates skin sparing effect of photons in head and neck cancer patients: a pilot study. Cancer Ther Oncol Int J.

[21] Acar H., Yazici O., Unal D. (2023). Bolus effect of immobilization mask during cyberknife treatment. J Med Biol Eng.

[22] Chang J, Tian Z, Lu W, - al, Huesa-Berral C, Juan-Cruz C, et al. Comparison of two thermoplastic immobilization mask systems in daily volumetric image guided radiation therapy for head and neck cancers. Biomed Phys Eng Express 2018;4:055007. 10.1088/2057-1976/AAD574.

[23] Kim S.H., Oh S.A., Yea J.W., Park J.W. (2019). Prospective assessment of inter- or intra-fractional variation according to body weight or volume change in patients with head and neck cancer undergoing radiotherapy. PLoS One.

[24] Lai Y.L., Yang S.N., Liang J.A., Wang Y.C., Yu C.Y., Su C.H. (2014). Impact of body-mass factors on setup displacement in patients with head and neck cancer treated with radiotherapy using daily on-line image guidance. Radiat Oncol.

[25] Ahn P.H., Chen C.C., Ahn A.I., Hong L., Scripes P.G., Shen J. (2011). Adaptive planning in intensity-modulated radiation therapy for head and neck cancers: single-institution experience and clinical implications. Int J Radiat Oncol Biol Phys.

[26] Wiant D., Squire S., Liu H., Maurer J., Hayes T.L., Sintay B. (2016). A prospective evaluation of open face masks for head and neck radiation therapy. Pract Radiat Oncol.

[27] Li G., Ballangrud A., Chan M., Ma R., Beal K., Yamada Y. (2015). Clinical experience with two frameless stereotactic radiosurgery (fSRS) systems using optical surface imaging for motion monitoring. J Appl Clin Med Phys.

[28] Al-Hallaq H.A., Cerviño L., Gutierrez A.N., Havnen-Smith A., Higgins S.A., Kügele M. (2022). AAPM task group report 302: surface-guided radiotherapy. Med Phys.

[29] Kang H.J., Grelewicz Z., Wiersma R.D. (2012). Development of an automated region of interest selection method for 3D surface monitoring of head motion. Med Phys.

[30] Gopan O., Wu Q. (2012). Evaluation of the accuracy of a 3D surface imaging system for patient setup in head and neck cancer radiotherapy. Int J Radiat Oncol Biol Phys.

[31] Bry V., Licon A.L., McCulloch J., Kirby N., Myers P., Saenz D. (2021). Quantifying false positional corrections due to facial motion using SGRT with open-face Masks. J Appl Clin Med Phys.

[32] Freislederer P., Batista V., Öllers M., Buschmann M., Steiner E., Kügele M. (2022). ESTRO-ACROP guideline on surface guided radiation therapy. Radiother Oncol.

[33] Carfagni M., Furferi R., Governi L., Santarelli C., Servi M., Uccheddu F. (2019). Metrological and critical characterization of the intel D415 stereo depth camera. Sensors.

[34] Ben Bouchta Y. GitHub - SpyGRT: Toolkit for fast SGRT application development n.d. https://github.com/Image-X-Institute/SpyGRT (accessed September 28, 2023).

[35] Zhou Q-Y, Park J, Koltun V. Open3D: A Modern Library for 3D Data Processing 2018 10.48550/arXiv.1801.09847.

[36] Chen Y., Medioni G. (1991). Object modeling by registration of multiple range images. Proc IEEE Int Conf Robot Autom.

[37] Barron JT. A General and Adaptive Robust Loss Function. 2019 IEEE/CVF Conference on Computer Vision and Pattern Recognition (CVPR) 2019; 2019-June:4326–34. 10.1109/CVPR.2019.00446.

[38] Shi K., Dipuglia A., Booth J., Alnaghy S., Kyme A., Keall P. (2020). Experimental evaluation of the dosimetric impact of intrafraction prostate rotation using film measurement with a 6DoF robotic arm. Med Phys.

[39] Alnaghy S., Kyme A., Caillet V., Nguyen D.T., O’Brien R., Booth J.T. (2019). A six-degree-of-freedom robotic motion system for quality assurance of real-time image-guided radiotherapy. Phys Med Biol.

[40] Lee S.K., Huang S., Zhang L., Ballangrud A.M., Aristophanous M., Cervino Arriba L.I. (2021). Accuracy of surface-guided patient setup for conventional radiotherapy of brain and nasopharynx cancer. J Appl Clin Med Phys.

[41] Stieler F., Wenz F., Shi M., Lohr F. (2013). A novel surface imaging system for patient positioning and surveillance during radiotherapy: a phantom study and clinical evaluation. Strahlenther Onkol.

[42] Covington E.L., Fiveash J.B., Wu X., Brezovich I., Willey C.D., Riley K. (2019). Optical surface guidance for submillimeter monitoring of patient position during frameless stereotactic radiotherapy. J Appl Clin Med Phys.

[43] Rasmussen K., Bry V., Papanikolaou N. (2020). Technical overview and features of the C-RAD catalyst^tm^ and sentinel^TM^ systems. Surface Guided Radiation Therapy.

[44] Shao H.C., Li Y., Wang J., Jiang S., Zhang Y. (2023). Real-time liver tumor localization via combined surface imaging and a single x-ray projection. Phys Med Biol.

