# Supplementary material for: The Remove-the-Mask Open-Source head and neck Surface-Guided radiation therapy system
Source: Phys Imaging Radiat Oncol. 2024 Jan 29;29:100541. doi: 10.1016/j.phro.2024.100541 (PMC10847032; doi:10.1016/j.phro.2024.100541)
Supplement: Supplementary data 1 [file mmc1.pdf]

Table S.1: Table of the accuracy (mean measurement error) and precision (standard deviation of error, noted SD in the table) of surface tracking for each rotational and translational axis for the traces reproduced on the UR16 and all 10 human traces.

	Mean Translation Error ± SD (mm)				Mean Rotation Error ± SD (def)		
	**LR**	**SI**	**AP**	**TDE***	**LR**	**SI**	**AP**
Female 1	1.1 ± 0.4	-0.3 ± 0.2	0.0 ± 0.1	1.2 ± 0.4	0.1 ± 0.1	-0.1 ± 0.1	0.6 ± 0.2
Female 2	-0.3 ± 0.1	0.5 ± 0.3	0.0 ± 0.1	0.6 ± 0.3	0.2 ± 0.1	0.1 ± 0.1	0.2 ± 0.2
Female 3	1.0 ± 0.5	0.5 ± 0.5	0.5 ± 0.3	1.3 ± 0.6	0.4 ± 0.3	-0.5 ± 0.2	0.0 ± 0.1
Female 4	0.8 ± 0.5	0.3 ± 0.9	0.1 ± 0.3	1.2 ± 0.7	0.0 ± 0.3	-0.2 ± 0.2	0.3 ± 0.1
Female 5	-0.4 ± 0.3	-0.6 ± 0.4	0.1 ± 0.1	0.9 ± 0.4	-0.3 ± 0.1	0.3 ± 0.2	0.4 ± 0.1
Male 1	-0.7 ± 0.7	-3.3 ± 1.2	0.7 ± 0.3	3.5 ± 1.3	-0.6 ± 0.3	0.2 ± 0.2	-0.5 ± 0.3
Male 2	-0.2 ± 0.3	-2.9 ± 1.1	1.1 ± 0.4	3.1 ± 1.2	-0.3 ± 0.1	0.1 ± 0.1	0.1 ± 0.1
Male 3	-1.6 ± 0.8	-0.3 ± 0.9	-0.3 ± 0.2	1.8 ± 0.9	0.2 ± 0.3	0.3 ± 0.1	-0.5 ± 0.2
Male 4	-0.2 ± 0.4	-0.4 ± 0.4	0.4 ± 0.2	0.7 ± 0.3	-0.1 ± 0.1	-0.1 ± 0.1	-0.3 ± 0.1
Male 5	-0.2 ± 0.3	0.6 ± 0.3	0.0 ± 0.2	0.7 ± 0.3	0.2 ± 0.1	-0.1 ± 0.1	-0.2 ± 0.1
Phantom S**	0.0 ± 0.4	0.1 ± 0.3	0.3 ± 0.4	0.5 ± 0.4	0.1 ± 0.2	0.2 ± 0.2	0.0 ± 0.2
Phantom 1	0.0 ± 0.3	0.0 ± 0.2	0.6 ± 0.3	0.8 ± 0.3	0.0 ± 0.1	0.2 ± 0.2	-0.2 ± 0.1
Phantom 2	0.0 ± 0.3	0.0 ± 0.2	0.5 ± 0.3	0.6 ± 0.3	0.0 ± 0.1	0.0 ± 0.1	0.1± 0.1
Phantom 3	-0.3 ± 0.2	-0.5 ± 0.2	1.5 ± 0.6	1.6 ± 0.6	0.1 ± 0.1	0 .1 ± 0.1	0.0 ± 0.1

** This column shows the Total Displacement Error which is the error in the measured Total Displacement Vector.*

*** This row shows the results from the phantom experiment where single axis translation and rotations were done one at a time successively.*
